# Supplementary material for: Association detection between multiple traits and rare variants based on family data via a nonparametric method
Source: PeerJ. 2023 Sep 26;11:e16040. doi: 10.7717/peerj.16040 (PMC10541022; doi:10.7717/peerj.16040)
Supplement: Supplemental Information 3 [file peerj-11-16040-s003.pdf]

Supplementary Materials

Table S1. Power comparisons of the Nonp-FAM and MAX-FAM methods for three ordinal traits at significance level $\alpha = 0.05$

RVs	Test	$\beta_1 = 0.8$ $\beta_2 = 0.4$ $\beta_3 = 0$	$\beta_1 = 0.6$ $\beta_2 = 0.3$ $\beta_3 = 0$	$\beta_1 = 0.4$ $\beta_2 = 0.2$ $\beta_3 = 0$	$\beta_1 = 0.2$ $\beta_2 = 0.1$ $\beta_3 = 0$
20	Nonp-FAM	0.9720	0.8990	0.6910	0.2920
(12 Causal)	MAX-FAM	0.1700	0.1100	0.1000	0.0690

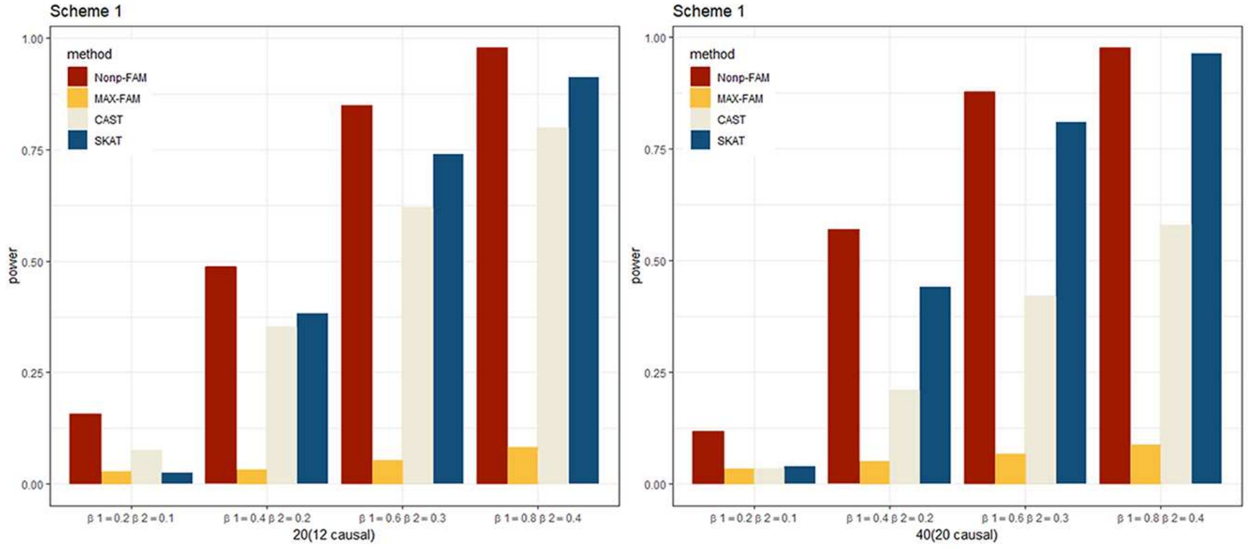

Figure S1. Power comparisons of the four methods for two binary traits in Scheme 1.

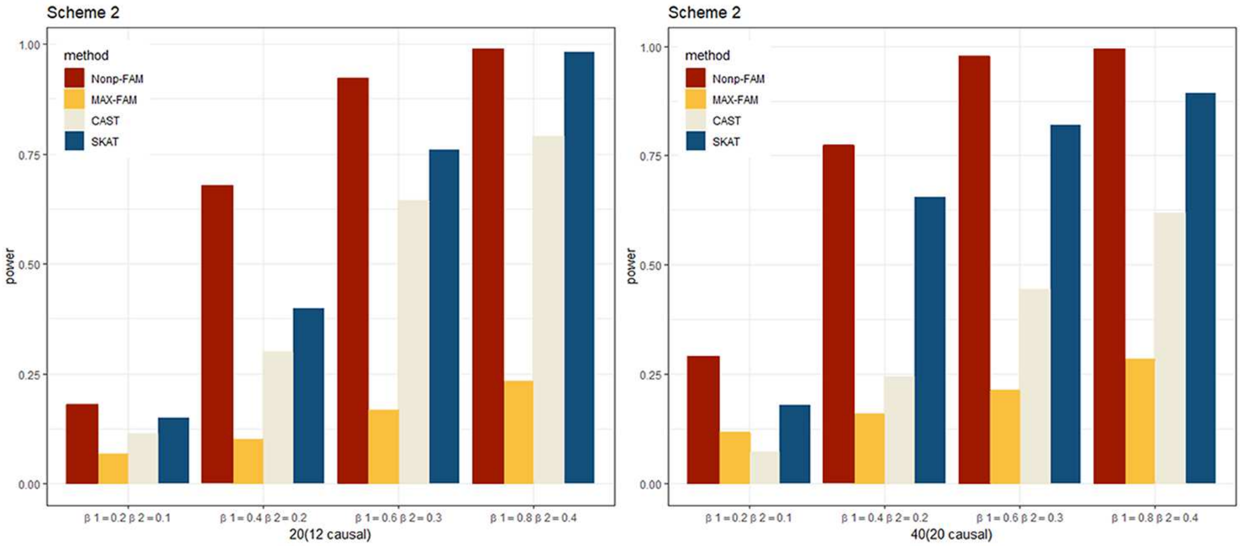

Figure S2. Power comparisons of the four methods for two ordinal traits in Scheme 2.

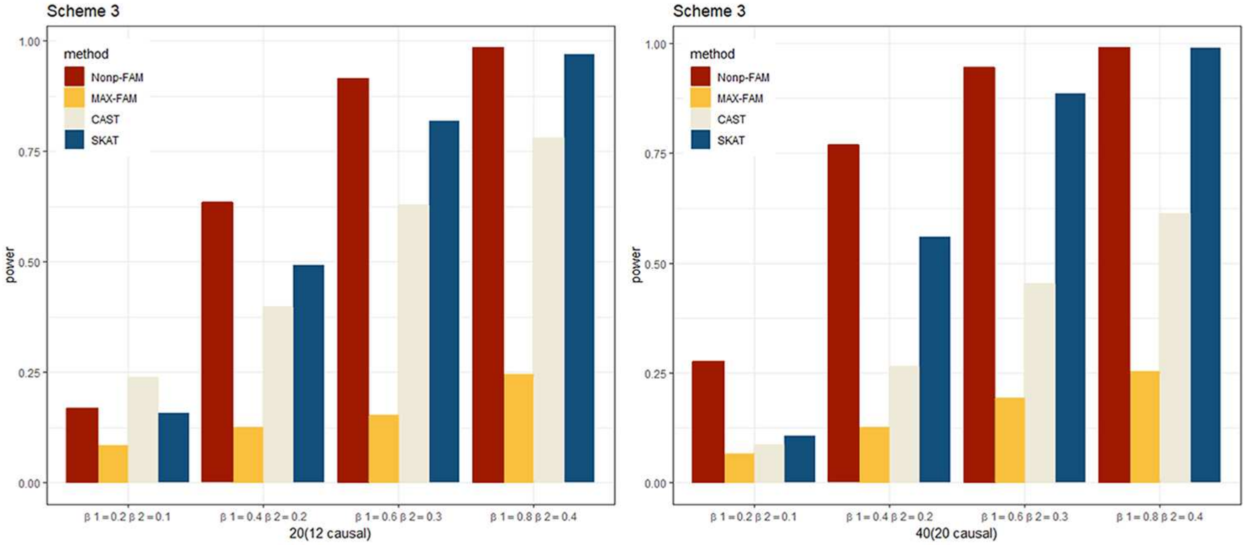

Figure S3. Power comparisons of the four methods for a mixture of binary and ordinal traits in Scheme 3.
