# Supplementary material for: Association detection between multiple traits and rare variants based on family data via a nonparametric method
Source: PeerJ. 2023 Sep 26;11:e16040. doi: 10.7717/peerj.16040 (PMC10541022; doi:10.7717/peerj.16040)
Supplement: Supplemental Information 4 [file peerj-11-16040-s004.pdf]

APPENDIX

The derivation process of $E(U|T)$ and $Var(U|T)$

Since $E(U|T) = \frac{2}{n-1}\sum_{i=1}^{n}\bar{F}_i E[\sum_{l=1}^{m}\log(n_{DG_{il}}n_{MG_{il}}n_{OG_{il}})|T]$, according to the independence of each rare variant locus, it is easy to obtain that

$$E(U|T) = \frac{2}{n-1}\sum_{i=1}^{n}\bar{F}_i\sum_{l=1}^{m}E[\log(n_{DG_{il}}n_{MG_{il}}n_{OG_{il}})|T],$$

where

$$\begin{aligned}
&E[\log(n_{DG_{il}}n_{MG_{il}}n_{OG_{il}})|T]\\
&= E[(\log n_{DG_{il}} + \log n_{MG_{il}} + \log n_{OG_{il}})|T]\\
&= E(\log n_{DG_{il}}|T) + E(\log n_{DG_{il}}|T) + E(\log n_{DG_{il}}|T)\\
&= P(DG_{il} = 0)\log n_{D0} + P(DG_{il} = 1)\log n_{D1} + P(DG_{il} = 2)\log n_{D2}\\
&\quad + P(MG_{il} = 0)\log n_{M0} + P(MG_{il} = 1)\log n_{M1} + P(MG_{il} = 2)\log n_{M2}\\
&\quad + P(OG_{il} = 0)\log n_{O0} + P(OG_{il} = 1)\log n_{O1} + P(OG_{il} = 2)\log n_{O2}\\
&= 3[\log n + 2(1-p_l)^2\log(1-p_l) + 2p_l(1-p_l)\log 2p_l(1-p_l) + 2{p_l}^2\log p_l]\\
&= 3[\log n + 2(1-p_l)\log(1-p_l) + 2p_l\log p_l + 2p_l(1-p_l)\log 2].
\end{aligned}$$

Besides, the genetic data of each family are independent, therefore

$$\begin{aligned}
Var(U|T) &= Var[\frac{2}{n-1}\sum_{l=1}^{m}\sum_{i=1}^{n}\bar{F}_i\log(n_{DG_{il}}n_{MG_{il}}n_{OG_{il}})|T]\\
&= \left(\frac{2}{n-1}\right)^2\sum_{i=1}^{n}\bar{F}_i\bar{F}_i^T Var[\sum_{l=1}^{m}\log(n_{DG_{il}}n_{MG_{il}}n_{OG_{il}})|T]\\
&= \left(\frac{2}{n-1}\right)^2\sum_{i=1}^{n}\bar{F}_i\bar{F}_i^T\sum_{l=1}^{m}Var[\log(n_{DG_{il}}n_{MG_{il}}n_{OG_{il}})|T],
\end{aligned}$$

where

$$\begin{aligned}
&Var[\log(n_{DG_{il}}n_{MG_{il}}n_{OG_{il}})|T]\\
&= E[\log^2(n_{DG_{il}}n_{MG_{il}}n_{OG_{il}})|T] - \{E[\log(n_{DG_{il}}n_{MG_{il}}n_{OG_{il}})|T]\}^2,
\end{aligned}$$

and

$$\begin{aligned}
&E[\log^2(n_{DG_{il}}n_{MG_{il}}n_{OG_{il}})|T]\\
&=E[(\log n_{DG_{il}} + \log n_{MG_{il}} + \log n_{OG_{il}})^2|T]\\
&= E[(\log^2 n_{DG_{il}} + \log^2 n_{MG_{il}} + \log^2 n_{OG_{il}} + 2\log n_{DG_{il}}\log n_{MG_{il}}\\
&\quad + 2\log n_{DG_{il}}\log n_{OG_{il}} + 2\log n_{MG_{il}}\log n_{OG_{il}})|T]\\
&=E(\log^2 n_{DG_{il}}|T) + E(\log^2 n_{MG_{il}}|T) + E(\log^2 n_{OG_{il}}|T)\\
&\quad + 2E(\log n_{DG_{il}}\log n_{MG_{il}}) + 2E[\log n_{OG_{il}}(\log n_{DG_{il}} + \log n_{MG_{il}})]\\
&= E(\log^2 n_{DG_{il}}|T) + E(\log^2 n_{MG_{il}}|T) + E(\log^2 n_{OG_{il}}|T)\\
&\quad + 2E(\log n_{DG_{il}}|T)E(\log n_{MG_{il}}|T) + 2E[\log n_{OG_{il}}(\log n_{DG_{il}}n_{MG_{il}})|T],
\end{aligned}$$

where

$$
\begin{aligned}
&E[\log n_{OG_{il}}(\log n_{DG_{il}} n_{MG_{il}})|T] \\
&= P(DG_{il}=0)P(MG_{il}=0)\log n_{O0}\log n_{D0}n_{M0} \\
&\quad +\tfrac{1}{2}P(DG_{il}=0)P(MG_{il}=1)\log n_{O0}\log n_{D0}n_{M1} \\
&\quad +\tfrac{1}{2}P(DG_{il}=1)P(MG_{il}=0)\log n_{O0}\log n_{D1}n_{M0} \\
&\quad +\tfrac{1}{2}P(DG_{il}=0)P(MG_{il}=1)\log n_{O1}\log n_{D0}n_{M1} \\
&\quad +\tfrac{1}{2}P(DG_{il}=1)P(MG_{il}=0)\log n_{O1}\log n_{D1}n_{M0} \\
&\quad +P(DG_{il}=0)P(MG_{il}=2)\log n_{O1}\log n_{D0}n_{M2} \\
&\quad +P(DG_{il}=2)P(MG_{il}=0)\log n_{O1}\log n_{D2}n_{M0} \\
&\quad +\tfrac{1}{4}P(DG_{il}=1)P(MG_{il}=1)\log n_{O0}\log n_{D1}n_{M1} \\
&\quad +\tfrac{1}{2}P(DG_{il}=1)P(MG_{il}=1)\log n_{O1}\log n_{D1}n_{M1} \\
&\quad +\tfrac{1}{4}P(DG_{il}=1)P(MG_{il}=1)\log n_{O2}\log n_{D1}n_{M1} \\
&\quad +\tfrac{1}{2}P(DG_{il}=1)P(MG_{il}=2)\log n_{O1}\log n_{D1}n_{M2} \\
&\quad +\tfrac{1}{2}P(DG_{il}=2)P(MG_{il}=1)\log n_{O1}\log n_{D2}n_{M1} \\
&\quad +\tfrac{1}{2}P(DG_{il}=1)P(MG_{il}=2)\log n_{O2}\log n_{D1}n_{M2} \\
&\quad +\tfrac{1}{2}P(DG_{il}=2)P(MG_{il}=1)\log n_{O2}\log n_{D2}n_{M1} \\
&\quad +P(DG_{il}=2)P(MG_{il}=2)\log n_{O2}\log n_{D2}n_{M2} \\
&=2\log^2 n[(1-p_l)^4+4p_l(1-p_l)^3+6{p_l}^2(1-p_l)^2+4{p_l}^3(1-p_l)+{p_l}^4] \\
&\quad +8(1-p_l)^2\log n\log(1-p_l)+8(1-p_l)^3\log^2(1-p_l)+8p_l(1-p_l)\log n \\
&\quad \log 2p_l(1-p_l)+8p_l(1-p_l)^2\log(1-p_l)\log 2p_l(1-p_l)+2p_l(1-p_l) \\
&\quad \log^2 2p_l(1-p_l)+8{p_l}^2\log n\log p_l+8{p_l}^2(1-p_l)\log p_l\log 2p_l(1-p_l) \\
&\quad +8{p_l}^3\log^2 p_l.
\end{aligned}
$$

Based on the above derivation, we have

$$
\begin{aligned}
&Var[\log(n_{DG_{il}}n_{MG_{il}}n_{OG_{il}})|T] \\
&= E[\log^2(n_{DG_{il}}n_{MG_{il}}n_{OG_{il}})|T]-E[\log(n_{DG_{il}}n_{MG_{il}}n_{OG_{il}})|T]^2 \\
&= 3\log^2 n\{1+2[(1-p_l)^4+4p_l(1-p_l)^3+6{p_l}^2(1-p_l)^2+4{p_l}^3(1-p_l)+{p_l}^4]\} \\
&\quad +36(1-p_l)^2\log n\log(1-p_l)+4(1-p_l)^2(2{p_l}^2-8p_l+9)\log^2(1-p_l) \\
&\quad +36p_l(1-p_l)\log n\log 2p_l(1-p_l)+2p_l(1-p_l)(5+4p_l(1-p_l))\log^2 2p_l(1-p_l) \\
&\quad +36{p_l}^2\log n\log p_l+4{p_l}^2(3+4p_l+2{p_l}^2)\log^2 p_l \\
&\quad +16p_l(2-p_l)(1-p_l)^2\log(1-p_l)\log 2p_l(1-p_l) \\
&\quad +16{p_l}^2(1-p_l)(1+p_l)\log p_l\log 2p_l(1-p_l)+16{p_l}^2(1-p_l)^2\log p_l\log(1-p_l) \\
&\quad -9\{\log^2 n+4(1-p_l)^4\log^2(1-p_l)+4{p_l}^2(1-p_l)^2\log^2 2p_l(1-p_l)+4{p_l}^4\log^2 p_l \\
&\quad +4(1-p_l)^2\log n\log(1-p_l)+4p_l(1-p_l)\log n\log 2p_l(1-p_l)+4{p_l}^2\log n\log p_l \\
&\quad +8p_l(1-p_l)^3\log(1-p_l)\log 2p_l(1-p_l)+8{p_l}^2(1-p_l)^2\log p_l\log(1-p_l) \\
&\quad +8{p_l}^3(1-p_l)\log p_l\log 2p_l(1-p_l)\} \\
&=4p_l(1-p_l)^2(10-7p_l)\log^2(1-p_l)+2p_l(1-p_l)(14{p_l}^2-14p_l+5)\log^2 2p_l(1-p_l) \\
&\quad +4{p_l}^2(1-p_l)(7p_l+3)\log^2 p_l+8p_l(1-p_l)^2(7p_l-5)\log(1-p_l)\log 2p_l(1-p_l) \\
&\quad +8{p_l}^2(1-p_l)(2-7p_l)\log p_l\log 2p_l(1-p_l)-56{p_l}^2(1-p_l)^2\log p_l\log(1-p_l).
\end{aligned}
$$
